# A Case Study Using DFT Calculation Aided Experimental Teaching of Organic Chemistry: Take the Ethyl Acetate Synthesis Experiment as an Example

**DOI:** 10.1021/acsomega.5c02491

**Published:** 2025-05-15

**Authors:** Dongdong Li, Jiawei Li, Changzhong Chen, Jun Chen, Xiaobing Lan

**Affiliations:** Hunan Provincial Key Laboratory of Xiangnan Rare-Precious Metals Compounds Research and Application, School of Chemistry and Environmental Science, 117769 Xiangnan University, Chenzhou 423000, China

## Abstract

Organic chemistry is an important basic course in the field of natural science, and its experimental teaching is also an important part of this course. How can students acquire practical ability in experimental practice within the limited class hours? Our organic chemistry experimental teaching team has developed a teaching model using density functional theory (DFT) calculation-aided experimental teaching of organic chemistry. In this paper, taking the ethyl acetate synthesis experiment as an example, we provided a case study using DFT calculation-aided experimental teaching of organic chemistry. The reaction mechanism of ethyl acetate was studied using Gaussian 09 software. The changes in reaction energy barrier and carbonyl carbon structure were also studied. Our results show that the rate-determining step is the nucleophilic addition. The reasonable raw material addition procedure is that the glacial acetic acid and anhydrous ethanol should first be added to a flask, followed by adding sulfuric acid slowly. DFT calculation can explain clearly the mechanism of esterification reaction in a graphic form, which is not only helpful for the students to better grasp the key points of the experiment but also beneficial for deeply understanding the esterification reaction. It provides some important guidance and reference for the teaching activities of organic chemistry in the university and high school.

## Graphical abstract

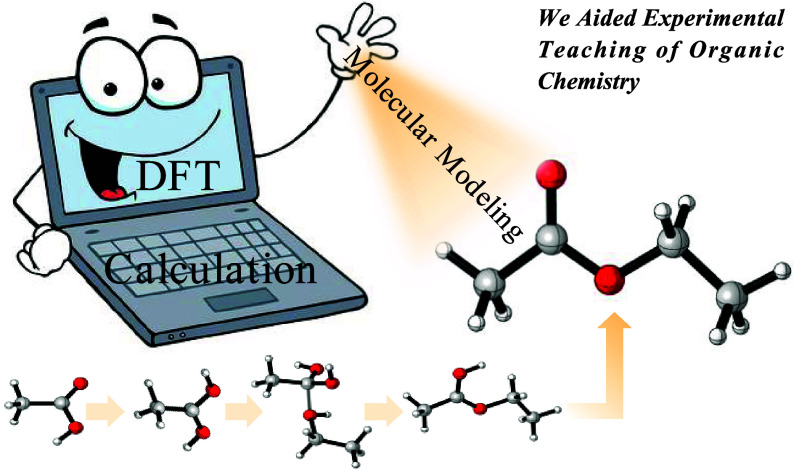

## 1 Introduction

Organic chemistry is a practical basic discipline, and its experiment teaching is an important part of this course and plays an irreplaceable role in strengthening students’ quality education and innovation ability.^1–7^ Although the ethyl acetate synthesis experiment is a classic and mature teaching experiment in organic chemistry,^8–11^ we still found that some students failed when they performed this experiment at Xiangnan University during the past three years (Table S1 in the Supporting Information). In our classroom, to begin the experiment, we repeatedly emphasized the details of the experiment. However, when the students began to operate the experiment independently, several failed in the experiment, resulting in a serious carbonization phenomenon (Figure 4). From our knowledge, students do not really understand the reaction mechanism, which is the main reason for the failed case.^12–14^ In this context, they cannot grasp the key points of the experiment. Density functional theory (DFT) calculation could be used to study the structures and properties of multielectron molecules in terms of the electron density, which is now widely used in organic chemistry scientific research.^15–19^ It can provide a visualization or graphical reaction path to help people understand the organic reaction mechanism, and it could well be used in teaching undergraduate organic chemistry.^20–24^ In an effort to introduce DFT calculation into the traditional undergraduate organic chemistry curriculum, a number of computational chemistry laboratory exercises have been developed and implemented.^25–29^

Esterification is a very important reaction in the course of organic chemistry in high school.^30–33^ In this stage, a written examination and score are mainly targeted. Thus, the students mechanically memorize that the acid takes off the hydroxyl group, the alcohol takes off the hydrogen, and then the ethyl acetate. All of the students showed familiarity with the reaction of glacial acetic acid with ethanol to produce ethyl acetate when they studied organic chemistry at Xiangnan University. However, they do not really understand how exactly this reaction happens step by step. Remember that acid dehydroxy and alcohol dehydrogenation to generate ethyl acetate is fine in high school, where examination questions are the main questions. However, it does not help in the content-heavy organic chemistry curriculum in higher education, which focuses on rapidly progressing through a range of reaction types and functional groups. It is far from enough for experimental teaching at a university.

In order to help students better understand the reaction mechanism of the esterification reaction between glacial acetic acid and ethanol for ethyl acetate synthesis, students also completed the experimental operation of this reaction within the limited class hours. Herein, we use Gaussian 09 software to perform DFT calculations on the reaction system of ethanoic acid and ethanol in the presence of an acid catalyst to generate ethyl acetate. The potential energy surface of glacial acetic acid with the ethanol reaction and the configuration change of carbonyl carbon during the reaction were investigated. The esterification reaction mechanism is explained visually and dynamically in graphic form. We also analyzed the influence of the order of adding materials for this experiment and the key operation points in the experiment. These results not only help students better grasp the operation points of this experiment but also facilitate a deeper understanding of the esterification reaction-related content. It has important guidance and reference significance for the teaching activities of organic chemistry courses in college and high school.

## 2 Materials and Methods

### 2.1 Computational Methods

All reactants, products, and transition states (ts) were fully optimized using the density functional theory at the B3LYP/6–31G­(d,p) level of theory.^34–36^ The dispersion correction with Grimme’s D3 method^37^ was taken into consideration in all calculations. Frequency analysis calculations were performed to characterize the structures at the minima (no imaginary frequency) or transition states (one imaginary frequency). The intrinsic reaction coordinate (IRC) calculations^38^ were carried out to verify that the predicted transition states connect the designated reactants and products. In order to obtain more accurate thermodynamics data, the single-point energies of all species were calculated by using the B3LYP/6–311++G­(d,p) method. All of the DFT calculations were performed using the Gaussian 09 program.^39^ The solvation effect of water was simulated by the SMD continuum solvent model.^40^ The 3D optimized structure figure in this paper is displayed by the CYLview visualization program.^41^ The bond length comparison of selected species and all of the optimized Cartesian coordinates of species involved in the reactions are available in the Supporting Information (SI). The partly selected experimental phenomenon is collected in Figure 1. The detailed numbers of students are collected in Table S1 in the SI.

**Figure 1:**
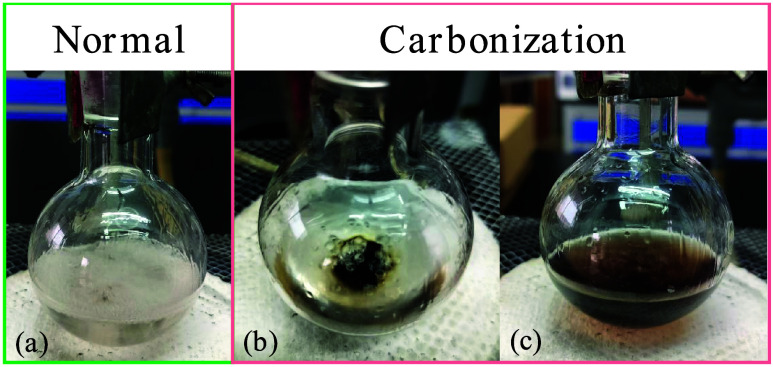
Experimental phenomenon. (a) Normal phenomenon. (b, c) Carbonization phenomenon.

### 2.2 Teaching Mode Implementation Process

The instructional design for the control group and experimental group is displayed in Figure 2 and is clearly implemented. If carbonization occurs during this experiment, we declare the experiment a failure. The control group received instruction with our traditional teaching mode (Figure 2a), while the experimental group engaged in our new teaching mode (Figure 2b). Before the experiment class, the class was divided into groups of 13–17 people. The preview content was arranged in advance, and the preview task is clear. We found that almost all students’ experiment preview reports just copied the experiment steps from the textbook. Thus, we deleted this step in our new teaching mode. We added an image presentation step in our new teaching mode. After displaying the energy profiles of glacial acetic acid with ethanol reaction and presenting the configuration of carbonyl carbon in the formation of ethyl acetate from glacial acetic acid and ethanol, the student began the experiment, and the experimental process can be carefully recorded.

**Figure 2:**
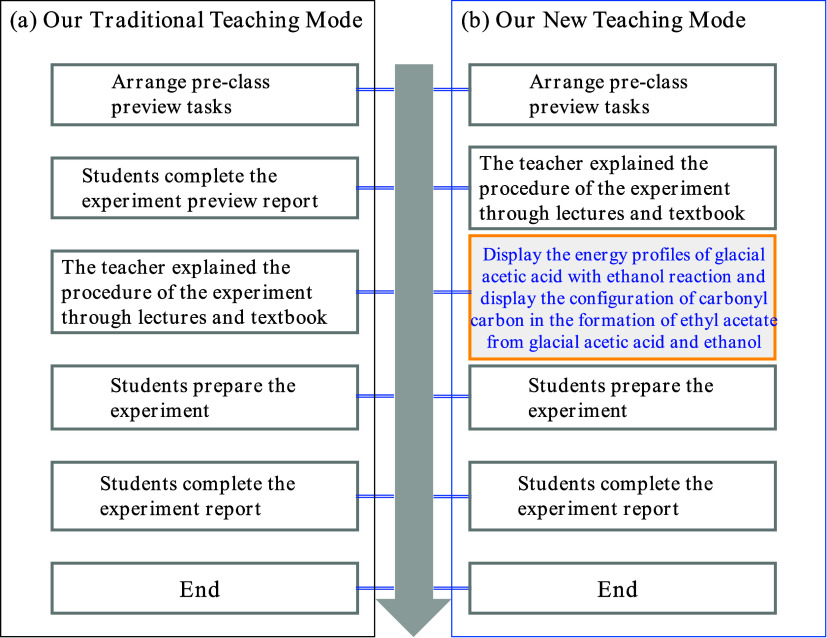
Teaching mode of the teaching process.

### 2.3 Effect Evaluation

In this study, learning efficacy is determined by assessing the success rate of students’ experimental implementations. As we all know, ethyl acetate synthesis is a classic and mature teaching experiment in the chemistry community. Therefore, in this case, the experiment is deemed unsuccessful if the carbonization phenomenon manifests during the reaction process.

## 3 Results and Discussion

### 3.1 Procedure of Ethyl Acetate Synthesis Experiment

In our organic chemistry teaching experiment course at Xiangnan University, the procedure of the ethyl acetate synthesis experiment is as follows (Figure 3). First, 9.5 mL of anhydrous ethanol and 6 mL of glacial acetic acid were added to a 50 mL round-bottom flask. Then, 2.5 mL of sulfuric acid was carefully added. After mixing well, an amount of zeolite was added and the flask was slowly heated to keep the reflux for 0.5 h. Second, heating was stopped. When the reactants in the flask had cooled slightly, the reflux device was changed into a distillation device, and the receiving flask was cooled down with cold water. Finally, when the resultant ethyl acetate was heated and distilled until the volume of the distilled liquid was about half of the total volume of the reactants, the reaction was stopped.

**Figure 3:**
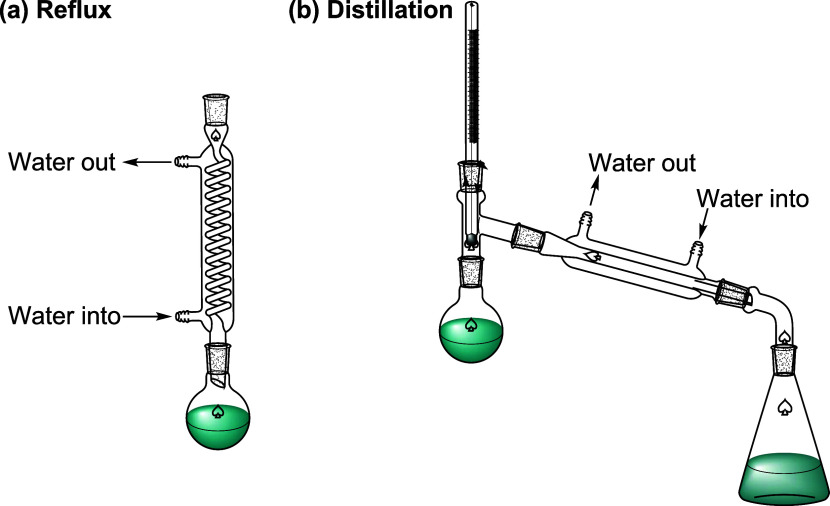
Diagram of the ethyl acetate synthesis experiment: (a) reflux and (b) distillation.

### 3.2 Design and Participants of the Study

As a classic teaching experiment in basic organic chemistry, we found that our procedure was very similar to that of the other textbooks. Thus, there is no problem with the procedure of the ethyl acetate synthesis experiment. However, in 2020, we found that some students failed, with the carbonization phenomenon occurring, when the students did this experiment. Therefore, our organic chemistry experimental teaching team decided to develop a teaching model using DFT calculation-aided experimental teaching of organic chemistry. A total of 411 students majoring in pharmacy at Xiangnan University in 2021, 2022, and 2023 batches were selected as participants (for more details, see Table S1 in the SI). Of these, class 3 adopted traditional teaching methods as the control group, and class 1 and class 2 adopted a new teaching mode as the experimental group. Both groups received the same organic chemistry course taught by the same faculty. The control group and experimental group of participants received different teaching modes. The control group consisted of students who received ethyl acetate synthesis experiment education through conventional lecture-based teaching methods, while the experimental group received instruction using DFT calculation-aided teaching strategies centered around the mechanism of esterification reaction in a graphic form. The research was conducted in the same classroom. We used the rate of carbonization to measure the learning outcomes of both groups; thus, we were able to determine the effectiveness of the new teaching strategies. The results are shown in Figure 4. For more details, see Table S1. As for the control group, the average carbonization rate is 8%. However, only 2% or no carbonization rate occurs in the experimental group. These results indicated that a low carbonization rate would be attributed to DFT calculation-aided teaching strategies.

**Figure 4:**
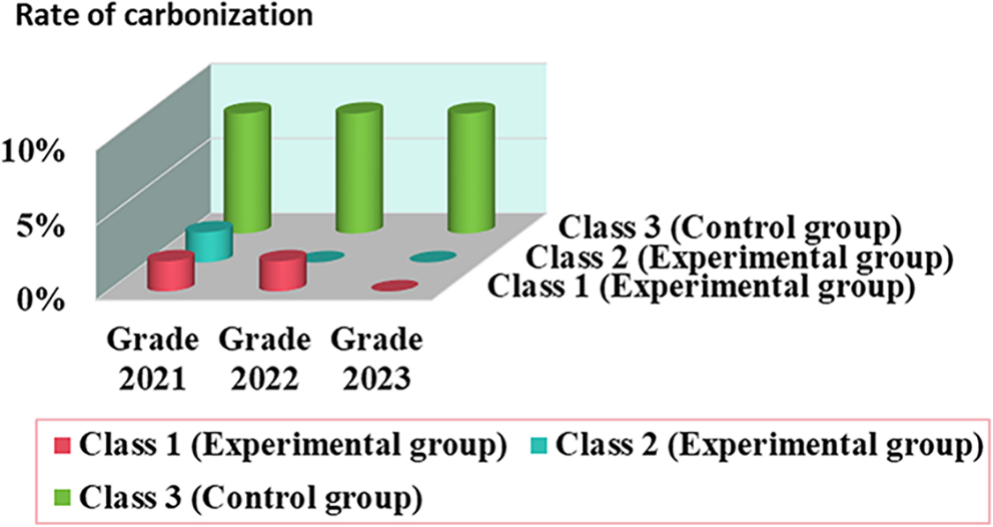
Rate of carbonization of the control group and the experimental group.

### 3.3 Reaction Model and Mechanism of Ethyl Acetate Synthesis

The reaction formula of glacial acetic acid (**1a**) and ethanol (**2a**) to form ethyl acetate (**3a**) under the catalysis of acid is shown in Figure 5a. Glacial acetic acid breaks the acyl-oxygen bond and loses the hydroxyl group, and alcohol removes the hydrogen group to form ethyl acetate. Generally, the reaction mechanism of this reaction is shown in Figure 5b. We can clearly see from the reaction mechanism that the reaction must be carried out with an acid proton catalyst. Sulfuric acid is added to catalyze the esterification reaction. Therefore, in principle, the reaction should be to first add glacial acetic acid, then add sulfuric acid, and finally ethanol. However, sulfuric acid is usually concentrated. If glacial acetic acid and concentrated sulfuric acid are added together first, glacial acetic acid will have a certain inhibitory effect on the ionization of concentrated sulfuric acid so that concentrated sulfuric acid cannot well ionize H^+^, resulting in poor catalytic performance. On the other hand, adding sulfuric acid to the solution system will release a lot of heat, which poses a certain risk. Therefore, a better choice should be to add acetic acid and ethanol first and mix them well. Then, slowly add sulfuric acid, which can not only reduce the inhibition of acetic acid on concentrated sulfuric acid ionization but also reduce the risk of concentrated sulfuric acid dilution caused by a large amount of heat released.

**Figure 5:**
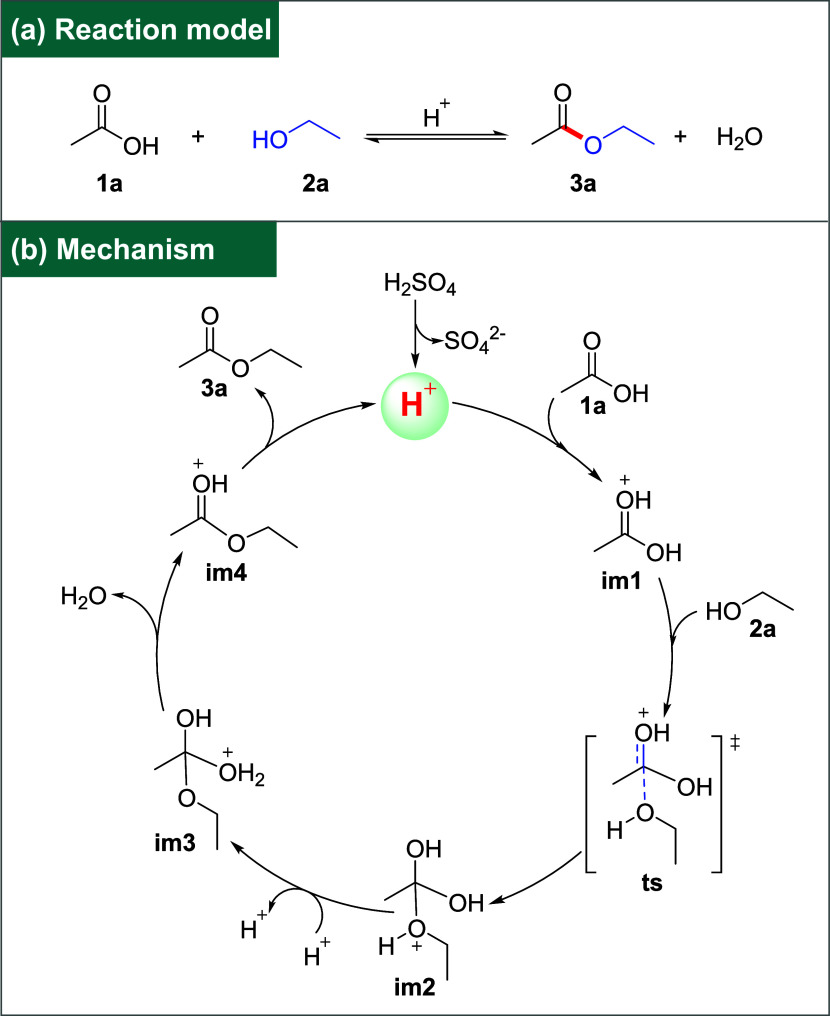
Reaction model and mechanism are cyclic: (a) reaction model and (b) mechanism.

### 3.4 Energy Profiles of Glacial Acetic Acid with Ethanol Reaction

We used the DFT theory to calculate the potential energy of the reaction of glacial acetic acid and ethanol to produce ethyl acetate, and the results are shown in Figure 6. First, glacial acetic acid **1a** is protonated to form the intermediate **im1**. Then, **im1** undergoes a nucleophilic addition with ethanol, forming intermediate **im2** through transition state **ts**. The transition state **ts** has an energy barrier of 19.6 kcal/mol, which is the highest in the reaction process. Therefore, the key rate-determining step in the esterification reaction mechanism is acid protonation, followed by the nucleophilic addition of alcohol. The intermediate **im2** forms the intermediate **im3** through proton transfer, and then the intermediate **im3** is dehydrated to obtain **im4**. Finally, the deprotonation of **im4** produces the product ethyl acetate (**3a**) and completes the reaction cycle. Using this reaction potential energy diagram in experimental teaching, students can clearly understand the energy change of the reaction between glacial acetic acid and ethanol to produce ethyl acetate under the catalysis of acid, and deeply understand the importance of catalyst acid proton in this reaction. Moreover, the reaction thermodynamics have also been analyzed (Figure S1 in the SI). Especially, the electrostatic potential surfaces (EPS) of **1a** and **im1** were well compared (Figure S2 in the SI). We can clearly see that **im1** has a higher positive charge than **1a**, which means that **im1** is more prone to nucleophilic addition reactions compared to **1a**. Thus, the protonation of glacial acetic acid **1a** to form intermediate **im1** plays a crucial role in this reaction.

**Figure 6:**
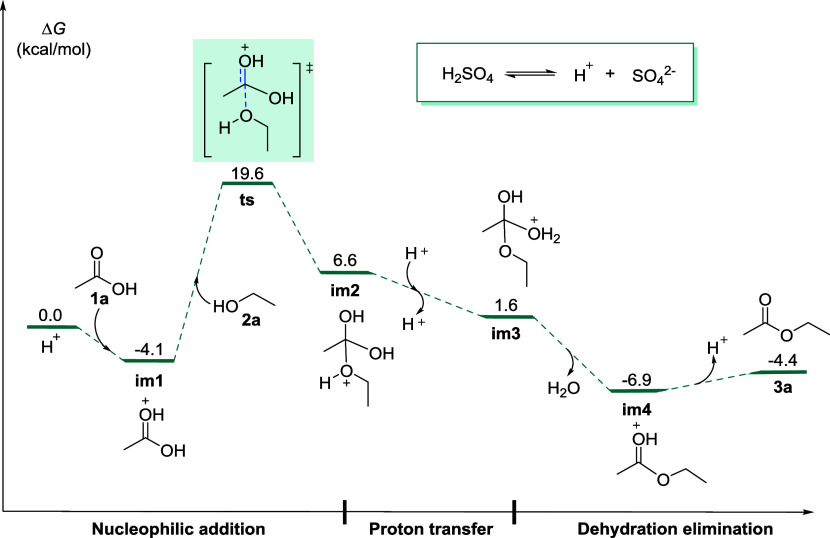
Energy profiles of glacial acetic acid with ethanol reaction.

### 3.5 Configuration of Carbonyl Carbon in the Formation of Ethyl Acetate from Glacial Acetic Acid and Ethanol

We investigated the changes in the configuration of the carbonyl carbon atom during the formation of ethyl acetate from acetic acid and ethanol using computational models (Figure 7). From Figure 7, we can clearly see that the configuration of the carbonyl carbon atom does not change before the transition state **ts** is formed and remains in a sp^2^-hybridized configuration. However, from the transition state **ts** onward, the carbonyl carbon atom gradually distorts and gradually transforms toward a tetrahedral sp^3^-hybridized configuration of a trigonal pyramidal structure. When it becomes intermediate **im3**, the carbonyl carbon atom is completely transformed into a sp^3^-hybridized trigonal pyramidal configuration. However, when **im3** loses water to form intermediate **im4**, the carbonyl carbon atom begins to gradually return to an sp^2^-hybridized configuration. When **im4** loses a proton to form ethyl acetate, the carbonyl carbon atom is completely transformed into an sp^2^-hybridized configuration. Using this diagram of the configuration change of the carbonyl carbon atom in the preparation of ethyl acetate experiment, students can clearly see the changes in the configuration of the carbonyl carbon atom during the formation of ethyl acetate from acetic acid and ethanol in the acid-catalyzed reaction, which is beneficial for deepening their understanding and recognizing the reaction process.

**Figure 7:**
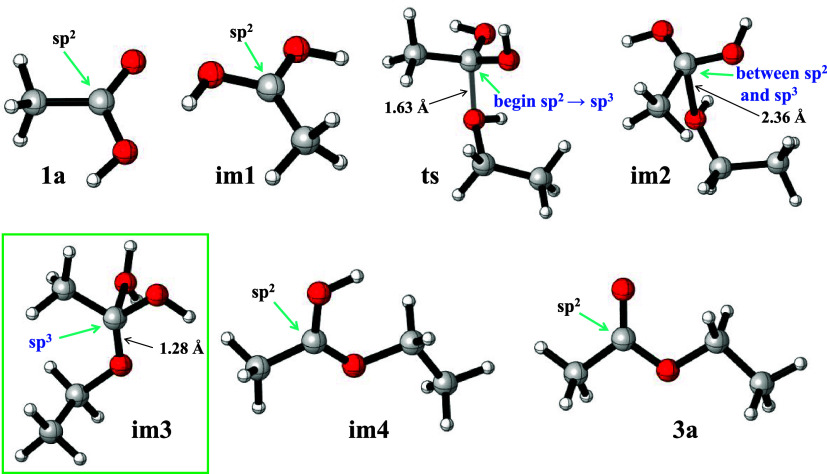
Configuration of carbonyl carbon in the formation of ethyl acetate from glacial acetic acid and ethanol.

## 4 Conclusions

In summary, based on the density functional theory, the reaction mechanism of acid-catalyzed ethanoic acid and ethanol to form ethyl acetate was studied using DFT computational means. The structures of reactants, intermediates, transition states, and products were optimized and analyzed for relative energies, and an energy change diagram of the reaction path was obtained. The results show that the key rate-determining step in the esterification reaction is protonation of the acid and nucleophilic addition of the alcohol. The appropriate feeding order for the preparation of ethyl acetate is found to be the addition of an ethanoic acid and ethanol mixture first, followed by the addition of concentrated sulfuric acid slowly. The kinetics of the reaction were studied by following the changes in the conformation of the carbonyl carbon atom. The reaction mechanism was quantitatively explained. These results not only help deepen students’ understanding of the preparation of ethyl acetate experiment, improve students’ understanding of the key points of the experiment, increase students’ interest in learning, further improve the success rate of the experiment, but also benefit the in-depth understanding of the related content of esterification reaction, which has important guidance and reference for organic chemistry teaching activities in universities and high schools.

## Supplementary Material



## Data Availability

Data are contained within the article.

## Notes

Optimized Cartesian coordinates of intermediates and transition states involved in Figures S6 and S7, Table S1, and Figures S1 and S2 (PDF)
