# Supplementary material for: A Case Study Using DFT Calculation Aided Experimental Teaching of Organic Chemistry: Take the Ethyl Acetate Synthesis Experiment as an Example
Source: ACS Omega. 2025 May 15;10(20):20922–8. doi: 10.1021/acsomega.5c02491 (PMC12120623; doi:10.1021/acsomega.5c02491)
Supplement: Supplementary file 1 [file ao5c02491_si_001.pdf]

Supporting Information

A Case Study Using DFT Calculation Aided Experimental Teaching of Organic Chemistry: Take the Ethyl Acetate Synthesis Experiment as an Example

Dongdong Li, Jiawei Li, Changzhong Chen, Jun Chen and Xiaobing Lan*

Hunan Provincial Key Laboratory of Xiangnan Rare-Precious Metals Compounds Research and Application, School of Chemistry and Environmental Science, Xiangnan University, Chenzhou 423000, China

E-mail: xblan@xnu.edu.cn

Table of Contents

1. Table S1. The details number of students and other information.............................1

2. Thermodynamic analysis of the esterification between glacial acetic acid and ethanol...1

3. The electrostatic potential surfaces of **1a** and **im1**..2

4. Optimized Cartesian coordinates...2

1. Table S1. The details number of students and other information

Table S1. The details number of students and other information

Entry	Grade	Class	Number of students	Number of carbonization	Rate of carbonization[c]
1[a]	2021	1	46	1	2%
2[a]	2021	2	49	1	2%
3[b]	2021	3	49	4	8%
4[a]	2022	1	39	1	2%
5[a]	2022	2	43	0	0%
6[b]	2022	3	39	3	8%
7[a]	2023	1	50	0	0%
8[a]	2023	2	47	0	0%
9[b]	2023	3	49	4	8%
Total	3	9	411	14	3%[d]

[a]Experimental group. [b]Control group. [c]Rate of carbonization=Number of carbonization/Number of students. [d]The average carbonization rate.

2. Thermodynamic analysis of the esterification between glacial acetic acid and ethanol

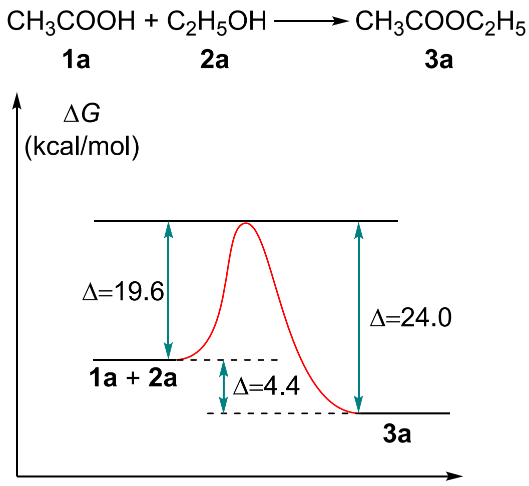

Figure S1. The reaction thermodynamics of glacial acetic acid with ethanol reaction.

3. The electrostatic potential surfaces of 1a and im1

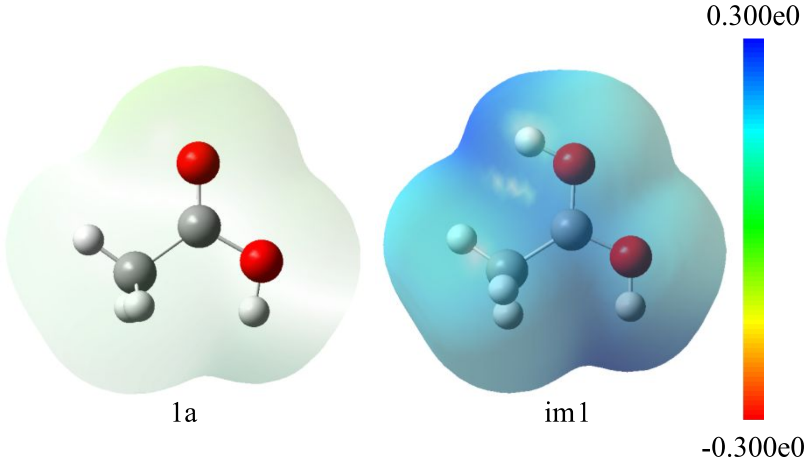

Figure S2. The electrostatic potential surfaces of **1a** and **im1**.

4. Optimized Cartesian coordinates

1a

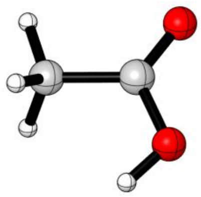

0 1

C	-0.13364400	0.13796800	0.00008200
O	-0.69748600	1.20122700	0.00003400
C	1.37534400	-0.04088700	-0.00003000
H	1.85193600	0.93846400	0.00017600
H	1.69684000	-0.60040300	0.88564100
H	1.69653100	-0.59988000	-0.88613200
O	-0.85617900	-1.01821900	-0.00014100
H	-0.26617700	-1.78472900	0.00086300

2a

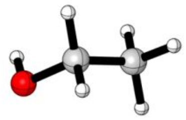

0 1

O	1.23942300	-0.26043600	-0.10814300
H	1.24403500	-0.89988400	0.61592900
C	0.08546600	0.55725100	0.04668000
H	0.12750500	1.28115200	-0.77426000
H	0.12537400	1.13488800	0.98549000
C	-1.21516300	-0.23999300	-0.02187100
H	-1.27768900	-0.95785500	0.80540800
H	-2.08678100	0.42048300	0.04462100
H	-1.26964100	-0.79884700	-0.96089200

3a

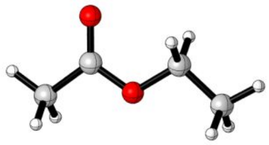

0 1

C	-2.13590000	0.89077800	0.00003000
H	-2.04334500	1.53213200	0.88148400
H	-3.10484900	0.39272200	-0.00017300
H	-2.04313000	1.53249900	-0.88113400
C	-1.03787100	-0.14681500	-0.00000800
O	-1.20108000	-1.34766200	0.00010400
O	0.17891000	0.44375400	-0.00015600
C	1.31185400	-0.45519300	-0.00015600
H	1.25309300	-1.10149600	-0.88235100
H	1.25285200	-1.10182200	0.88178200
C	2.56436900	0.40039900	0.00015100

H	3.45336700	-0.23795500	0.00009400
H	2.59742400	1.04029600	-0.88638000
H	2.59723600	1.03987500	0.88699200

H_3O^+

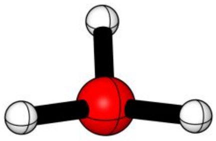

1 1

O	0.00000000	0.00000000	0.07010100
H	0.00000000	0.94749600	-0.18693600
H	-0.82055600	-0.47374800	-0.18693600
H	0.82055600	-0.47374800	-0.18693600

H_2O

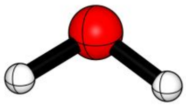

0 1

O	0.00000000	0.00000000	0.11884100
H	0.00000000	-0.76012300	-0.47536500
H	0.00000000	0.76012300	-0.47536500

im1

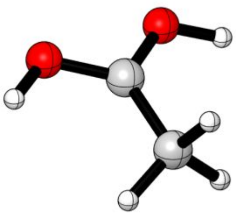

1 1

C	-0.08244600	-0.00123200	-0.01118400
O	-0.82527300	-1.04710300	0.00261800
C	1.40559400	-0.03464700	-0.00202500
H	1.79053500	-0.99271000	-0.35746700
H	1.81437900	0.77101200	-0.61862600
H	1.74521600	0.11839700	1.03210100
O	-0.76558800	1.08576000	0.00239900
H	-0.22746000	1.89762000	-0.00829400
H	-0.33467400	-1.88829800	-0.00860000

im2

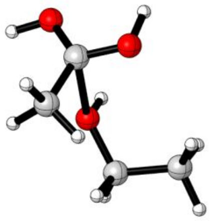

1 1

O	1.06256300	-1.41619400	0.20823300
C	1.24072500	0.69241600	1.28806400
H	0.48590000	0.29748800	1.96708300
H	2.21612400	0.65824100	1.79091500
H	1.00569000	1.72110100	1.01378400
O	1.90635600	0.19089900	-1.02472700
O	-0.72124700	0.70893200	-0.68645800
C	-1.96210200	0.66086200	0.06550700
H	-2.67033400	1.36953500	-0.37898800
H	-1.70687200	1.03708100	1.06015600

C	-2.54099400	-0.74323800	0.13255900
H	-1.83475100	-1.44001100	0.59420100
H	-3.45999100	-0.74509800	0.72605500
H	-2.79447400	-1.11418100	-0.86688400
H	-0.91684600	0.43856000	-1.59559500
C	1.32958100	-0.16319800	0.08151100
H	1.24006600	-1.92416800	-0.60727300
H	2.05085800	1.15130700	-1.08567400

im3

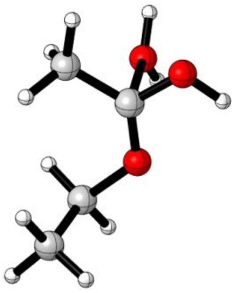

1 1

O	-1.35303000	1.34862200	-0.77404300
C	-0.54897100	0.81056000	1.38842100
H	0.43178100	1.05655100	1.80210700
H	-1.27571900	1.57550400	1.65895600
H	-0.86939200	-0.15841400	1.78386700
O	-1.89772800	-1.55469100	0.08600900
O	0.39489900	0.03546000	-0.75123500
C	1.43644800	-0.81448700	-0.10162400

H	1.54546700	-1.63082200	-0.81509200
H	1.02024300	-1.20191600	0.82907700
C	2.71257600	-0.01982900	0.07035300
H	2.59050100	0.81011500	0.77149300
H	3.48297200	-0.68676300	0.46896200
H	3.06176400	0.37133600	-0.88792800
C	-0.47823300	0.68983800	-0.08849800
H	-1.25953300	1.18699700	-1.73314700
H	-1.79835100	-2.40057200	-0.36948700
H	-2.81377600	-1.55362800	0.39342400

im4

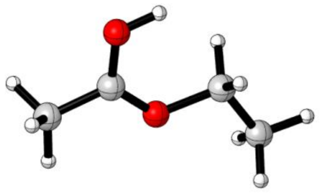

1 1

C	-2.11796700	-0.96017700	0.00008900
H	-1.73528000	-1.97939900	-0.00049800
H	-2.74428900	-0.78083800	-0.88084900
H	-2.74358200	-0.78149500	0.88164500
C	-1.00225500	0.01330100	-0.00003800
O	-1.32114200	1.26952900	0.00016000
O	0.19657400	-0.42135500	-0.00033600
C	1.40058200	0.47361700	-0.00016400

H	1.33500300	1.08881000	0.90334700
H	1.33543000	1.08856200	-0.90387100
C	2.61224800	-0.42744800	0.00023900
H	3.50825200	0.20014300	0.00030100
H	2.63047600	-1.05670600	0.89253100
H	2.63086500	-1.05698300	-0.89185700
H	-0.57598600	1.89674800	-0.00010300

ts1

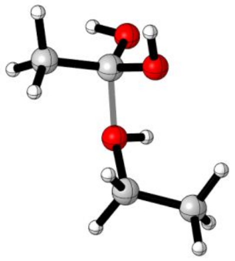

1 1

C	0.96787300	-0.12618600	0.06853900
O	0.41652900	-0.72181800	1.15436000
C	1.66825400	1.19561400	0.27620200
H	1.04249700	1.87812400	0.85288100
H	2.59255000	1.01263200	0.83307600
H	1.91657700	1.66083800	-0.68021300
O	1.57625300	-1.02972400	-0.72674900
H	2.17568000	-0.60604000	-1.36093400
O	-0.36407300	0.24203400	-0.79500000

H	-0.56141500	-0.56736300	-1.30636900
C	-1.61522800	0.74974000	-0.11284500
H	-1.98093400	1.52372000	-0.78860000
H	-1.25269900	1.19742200	0.81135400
C	-2.59312800	-0.37705400	0.12187100
H	-2.16846800	-1.13695500	0.78073300
H	-3.48784500	0.03498800	0.59892400
H	-2.91289100	-0.83632600	-0.81981100
H	1.04066000	-0.73767000	1.89547400
